# Experimental study of the dispersion of cough-generated droplets from a person going up- or downstairs

**DOI:** 10.1063/5.0073880

**Published:** 2022-01-03

**Authors:** Hongping Wang, Zhaobin Li, Yi Liu, Lixing Zhu, Zhideng Zhou

**Affiliations:** 1 The State Key Laboratory of Nonlinear Mechanics, Institute of Mechanics, Chinese Academy of Sciences, Beijing 100190, China; 2 School of Engineering Science, University of Chinese Academy of Sciences, Beijing 100049, China

## Abstract

The dispersion of cough-generated droplets from a person going up- or downstairs was investigated through a laboratory experiment in a water tunnel. This experiment was carried out with a manikin mounted at inclination angles facing the incoming flow to mimic a person going up or down. Detailed velocity measurements and flow visualization were conducted in the water tunnel experiments. To investigate the influence of the initial position on the motion of particles, a virtual particle approach was adopted to simulate the dispersion of particles using the measured velocity field. Particle clustering, which is caused by the unsteadiness of the flow, was observed in both flow visualization and virtual particle simulation. For the case of going upstairs, particles are concentrated below the person’s shoulder and move downward with a short travel distance. For the case of going downstairs, particles dispersing over the person’s head advect over for a long distance. We also found that the motion of the particles is closely related to the initial position. According to the results in this study, suggestions for the prevention of respiratory infectious disease are made.

## I. INTRODUCTION

A global pandemic infectious respiratory disease, coronavirus disease 2019 (COVID-19), is threatening human life and health. Since the outbreak of COVID-19, fluid droplets expelled from the respiratory tract have attracted much attention because the dispersion of the infectious virus in droplets generated by talking, coughing, and sneezing is one of the major routes of virus transmission (Wells, 1934; Xie *et al.*, 2009; Bourouiba *et al.*, 2014; Asadi *et al.*, 2019; Smith *et al.*, 2020; Stadnytskyi *et al.*, 2020; Morawska and Milton, 2020; and Hossain and Faisal, 2021). The expelled droplets have a broad range of sizes, from less than a micrometer to a thousand micrometers, resulting from atomization and evaporation (Xie *et al.*, 2009; Han *et al.*, 2013; and Wang *et al.*, 2020b). The motion of the droplets is determined by their diameters and initial velocities, cough airflow, and ambient airflow (Wang *et al.*, 2020a). Large droplets mainly settle downward, while small droplets can follow the motion of the airflow for more than 15 minutes (Abuhegazy *et al.*, 2020). Khosronejad *et al.* (2020) carried out a high-fidelity large-eddy simulation (LES) to study saliva transport during coughing under both indoor (stagnant background air) and outdoor (unidirectional mild breeze) conditions. Their results indicate that the leakage saliva through the facial mask under outdoor conditions can travel long distances by the ambient turbulent flow. Healthy persons may become infected by inhaling airborne droplets of the virus with even a short exposure. Therefore, it is critical to understand droplet transmission under different typical flow conditions in real life for modeling and controlling the epidemics. There have been numerous studies investigating droplet transmission in various environments, such as in a toilet (Li *et al.*, 2020a), in an elevator (Dbouk and Drikakis, 2021; Sen, 2021), in a dental clinic (Li *et al.*, 2021a; Komperda *et al.*, 2021), in a restaurant (Liu *et al.*, 2021), in a classroom (He *et al.*, 2021; Narayanan and Yang, 2021), and in the public transport (Zhang *et al.*, 2021; Talaat *et al.*, 2021). Most of these studies adopted numerical simulations to obtain the droplet motions, where the flow fields were solved using direct numerical simulation (Fabregat *et al.*, 2021a; b), LES (Khosronejad *et al.*, 2020) or Reynolds-averaged Navier–Stokes (RANS) equations (Dbouk and Drikakis, 2021; Li *et al.*, 2020a; 2020b; and Hossain and Faisal, 2021) coupled with Lagrangian particle tracking methods. Experiments were also carried out to study the flow dynamics of the droplets expelled by a sneeze (Bahl *et al.*, 2020) and to investigate the performance of face shields and masks on the prevention of droplet spread (Verma *et al.*, 2020a; 2020b; and Arumuru *et al.*, 2021).

Recently, Li *et al.* (2021b) numerically investigated the dispersion of cough-generated droplets from a person going up or down on an escalator and studied the effects of the slope and speed of the escalator on droplet dispersion. They observed a “downwash” flow in the person’s wake for the case of the ascending escalator and an “upwash” flow for the person on the descending escalator. The difference in the wake structures behind the person has a significant impact on the dispersion of the droplets. The droplets from the descending person can travel a long distance at the height of the head, and a person following behind [even at the safe distance suggested by World Health Organization (2021)] is at risk of inhaling the droplets. In the above work, a RANS solver was employed to obtain the steady flow field, and a stochastic velocity model was used to study the influence of turbulent fluctuations on droplet motions. Alternatively, Wang *et al.* (2020b) proposed a continuous random walk model to simulate turbulent fluctuations in violent expiratory events. Few studies have investigated the influences of unsteady flow on the dispersion of respiratory fluid droplets.

In this paper, we carry out laboratory water tunnel experiments to investigate the droplet dispersion exhaled from a person going up- or downstairs, with the particular focus on elucidating the effects of flow unsteadiness on droplet dispersion. The rest of this paper is organized as follows: We first introduce the experimental setup in Sec. II. The results in terms of flow fields and particle dispersion are analyzed in Sec. III. A virtual particle approach is also adopted to study the effect of the initial position on the particle concentration. Finally, we offer discussions and conclusions on the particle dispersion for a person going up- or downstairs in Sec. IV. Suggestions for the prevention of respiratory infectious diseases are proposed based on the analyses of our results.

## II. EXPERIMENTAL SETUP

**FIG. 1.**
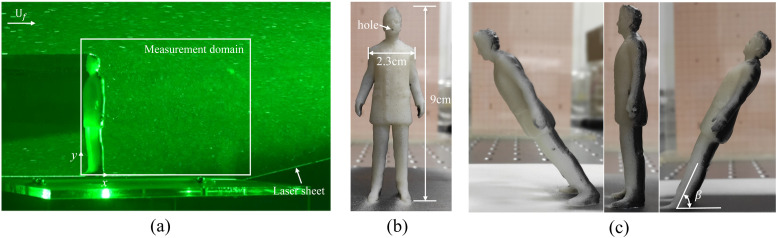
(a) Schematic diagram of the experimental setup; the incoming flow speed is *U*_*f*_. (b) Front view of the manikin used in the experiment. (c) Side view of the manikin. From left to right are the inclination angles *β* = 120°, 90°, and 60°, where *β* = 120° and 60° represent the person going up- and downstairs, respectively, while *β* = 90° corresponds to the case in which a person walks on flat ground.

**FIG. 2.**
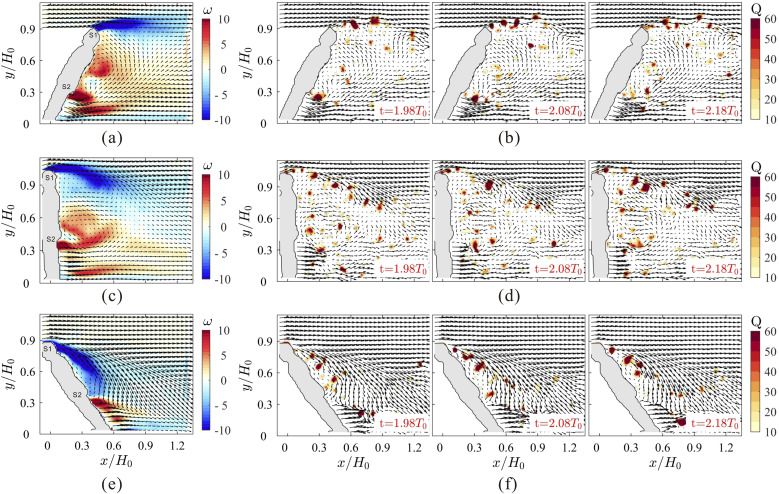
Mean flow fields (a), (c), and (e) and instantaneous flow fields (b), (d), and (f) with superimposition of vectors, one for every three vectors, are shown for clarity. The contours in (a), (c), and (e) represent the mean vorticity, and the contours in (b), (d), and (f) represent vortical structures identified by the *Q*-criterion. The nondimensional time interval in these figures corresponds to a physical time interval of 0.05 s.

In this study, laboratory experiments using manikin models were carried out in a water tunnel. The measurement domain covers the whole region behind the manikin, as shown in Fig. 1(a). The water tunnel, made of acrylic plates, has a test section with a cross section of 380 × 380 mm^2^ and a streamwise length of 800 mm. The free-stream velocity *U*_*f*_ is 180 mm/s with a turbulence intensity of ∼1.5%, which was measured using particle image velocimetry (PIV). As shown in Fig. 1, a human-shaped manikin model was mounted ∼27 cm downstream from the inlet of the test section. The manikin model has a height of 9 cm, denoted by *H*_0_. The Reynolds number based on the height of the manikin and incoming flow speed is ∼1.5 × 10^4^ (the temperature of water is ∼20°C), which is smaller than the practical Reynolds number of $\approx 10^{5}$ due to the limitation of the experimental equipment. Compared with the simulated results given in Fig. 2 from Li *et al.* (2021b), the flow pattern obtained by our experiments (see Fig. 2) is similar to their numerical results, although the Reynolds number is different. Therefore, the experimental fields are assumed to be equivalent to the real situation. The feet of the manikin were fixed on a flat plate, as shown in Fig. 1(a), which was raised ∼10 cm from the bottom wall of the water tunnel, so that the head of the manikin was approximately in the center of the cross section. Three manikin models were manufactured in a three-dimensional printer with white resin. The model was mounted facing the incoming flow at an inclination angle *β*, as sketched in Fig. 1(c). Three different inclination angles were considered in this study, i.e., *β* = 60°, 90°, and 120°, where *β* = 120° and 60° represent the person going up- and downstairs, respectively, while *β* = 90° corresponds to the case of a person walking on flat ground. Each model had a small hole (of diameter 1 mm) at the mouth and one hole at the feet (to connect with the hollow body). A lifted bottle was connected to the hole at the feet through a flexible tube. The bottle was filled with a mixture of water and fluorescent particles; the median diameter of the particles was 7 *μ*m and the density was 1500 kg/m^3^. A speed-control switch was installed on the tube. When the switch is opened, the fluorescent mixture driven by gravity is ejected into the flow field against the incoming flow from the mouth of the manikin. The average ejection speed was ∼33 cm/s, estimated from the flow and time. Note that the fluorescent particles were only used to qualitatively visualize the motion of ejected particles.

Two kinds of experiments are carried out to serve different purposes. One is flow visualization of the particle dispersion by using fluorescent particles ejected from the mouth of the manikin, and the other is flow field measurement behind the manikin by adopting PIV. For flow visualization, only the fluorescent particles are ejected into the water tunnel, and these particles are excited by a continuous 532 nm laser sheet with a thickness of 1 mm. The laser sheet is parallel to the sidewall and illuminates the flow field in the central plane of the manikin. The back of the manikins is painted with black flat lacquer to reduce the surface light reflection. The visualization images for particle dispersion are recorded by a high-speed camera (Fastcam SA8/15K-M2 with a resolution of 1280 × 1024 pixels^2^) equipped with a long-pass filter. For flow field measurement, the experimental configuration is similar to that of flow visualization, except that hollow glass microspheres with a diameter of 20–60 *μ*m are seeded in the water tunnel as tracers. Table I presents the parameters of the PIV experiments. The camera is placed perpendicularly to the sidewall of the tunnel, and the field of view is ∼15 × 12 cm^2^. The motion of the tracer is recorded at a frequency of 500 Hz for each case. An in-house PIV software with a three-pass window deformation iterative multigrid scheme is used to compute the velocity fields from the PIV images. The initial interrogation window is 96 × 96 pixels^2^, and the final interrogation window is 48 × 48 pixels^2^ with an overlap of 75%. The PIV velocity outliers are identified through the normalized median test and are replaced by the vectors obtained using linear interpolation of the neighboring vectors. The final velocity fields are smoothed by a two-dimensional Gaussian filter with a size of three grids. In the present work, the velocity and the length are nondimensionalized by the free-stream velocity *U*_*f*_ and the height of the manikin *H*_0_, respectively. Accordingly, the time is scaled by *H*_0_/*U*_*f*_.

**TABLE I.** The parameters of the PIV experiments.

Manikin height (cm)	Angle *β* (deg)	Flow speed (m/s)	Field of view (cm^2^)	Sampling frequency (Hz)	Image size (pixel^2^)
9	60	0.18	15 × 12	500	1280 × 1024
9	90	0.18	15 × 12	500	1280 × 1024
9	120	0.18	15 × 12	500	1280 × 1024

## III. RESULTS

### A. Flow field

We first examine the mean velocity fields for three inclination angles in Figs. 2(a), 2(c), and 2(e). The mean velocity fields agree well with the results of the RANS simulation in Li *et al.* (2020b, 2021b). Only the wake flow in the horizontal–vertical plane is analyzed since we focus on the motion of particles in the gravitational direction. When the manikin leans forward (*β* = 120°) to represent a person walking upstairs [see Fig. 2(e)], the flow separates at the head, indicated by *S*1, and reattaches at the hip *S*2, generating a separation or a recirculation region. For the inclination angle of 90°, representing the person walking on a flat ground, the flow separates at both locations *S*1 and *S*2, as shown in Fig. 2(c). Consequently, two large recirculation regions are formed behind the manikin, and the reattachment point is approximately at the back of the manikin body. When the inclination angle is decreased to 60° to simulate the person walking downstairs [Fig. 2(a)], flow separation mainly occurs at location *S*2 and the flow reattaches to the manikin body at approximately location *S*1. As a result, a vortical structure with positive spanwise vorticity is formed behind the manikin body. In summary, flow separation arises primarily at locations *S*1 and *S*2 for a person walking up- and downstairs, respectively. In Figs. 2(a), 2(c), and 2(e), a “downwash” mean velocity field behind the manikin can be seen when the flow separation occurs at *S*1, while an “upwash” mean velocity field is observed when the flow separation occurs at *S*2. This shows that the inclination angle has a distinct effect on the airflow behind a walking person, as evidenced in the simulation (Li *et al.*, 2021b).

The instantaneous vortical structures, rendered using the *Q*-criterion, are displayed in Figs. 2(b), 2(d), and 2(f) at three instants for each case. The *Q*-criterion is a vortex identification criterion estimated as the second invariant of the velocity gradient tensor (Hunt *et al.*, 1988), which indicates the region with vorticity larger than the strain. The vortices marked by the *Q*-criterion are mainly shed from locations *S*1 and *S*2. The vortices shedding from *S*1 are advected at the height of the head due to the “upwash” effect [see Fig. 2(b)] for *β* = 60°, whereas the vortical structures move toward the ground due to the “downwash” effect [see Fig. 2(c)] for *β* = 120°. No clear periodicity was observed in the vortex shedding given by the considerable three-dimensional effect.

### B. Dispersion of particles

**FIG. 3.**
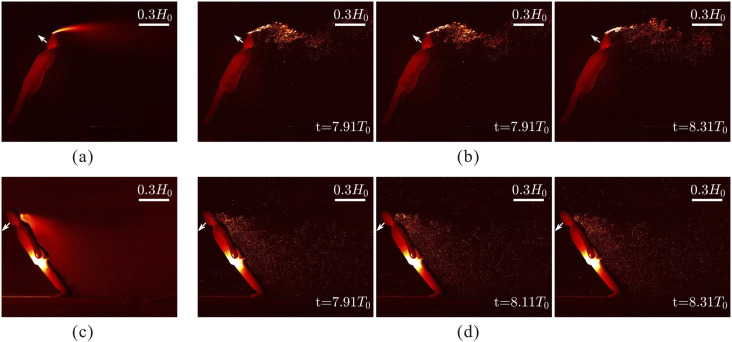
Visualization of the motion of particles expelled from the mouth of the manikin with inclination angles of 60° (a) and (b) and 120° (c) and (d). Panels (a) and (c) display the average intensity of particle fluorescence, and panels (b) and (d) show the instantaneous particle distribution at three times. The white arrows mark the ejection direction of the particles. The nondimensional time interval in these figures corresponds to a physical time interval of 0.1 s.

In addition to the transmission of large droplets, airborne transmission is also a critical route for the transmission of respiratory diseases. Aerosols with diameters smaller than 10 *μ*m can be generated via expiratory events or evaporation (Wang *et al.*, 2020a; Dbouk and Drikakis, 2020a; 2021; Morawska and Milton, 2020; Abuhegazy *et al.*, 2020; Liu *et al.*, 2020; Stadnytskyi *et al.*, 2020; and Mittal *et al.*, 2020). Given their small sizes, the aerosols are expected to follow the motion of airflow for a considerably long time. To investigate this scenario, fluorescent particles with a median diameter of 7 *μ*m were ejected from the mouth of the manikin to mimic small virus-laden respiratory fluid droplets expelled by coughing or sneezing. The response frequency of fluorescent particles is ∼250 kHz, which implies that the fluorescent particles can fully follow the flow motion. Therefore, the fluorescent particles in the water were used to simulate the airborne transmission of small droplets. The particle distribution is exhibited in Fig. 3, where the top and bottom rows show the results corresponding to the person going down- and upstairs, respectively. Figures 3(a) and 3(c) display the intensity of particle fluorescence averaged over all measured images, and Figs. 3(b) and 3(d) show the instantaneous particle distribution at individual times. The particle dispersion behind the manikin is different when the manikin is leaning forward or backward. For a person walking upstairs or ascending with an escalator [see Figs. 3(c) and 3(d)], most particles distribute below the manikin shoulder and move downward due to the reattachment circulation and the “downwash” velocity. The particles are spread more uniformly in space [see Fig. 3(d)], and the spatially local concentration of the particles decreases quickly and is expected to drop lower than a safe concentration quickly. For a person walking downstairs or descending on an escalator [see Figs. 3(a) and 3(b)], particles are advected over the head of the manikin. The particle distribution concentrates in a narrow band located at a height slightly higher than the head due to the “upwash” velocity. This particle band is elongated downstream, while the local concentration of the particles remains at a high level for a long distance. This suggests that a person of a similar height suffers a higher risk of infection when walking downstairs in the wake of an infectious person.

### C. Effect of the initial position on particle concentration

**FIG. 4.**
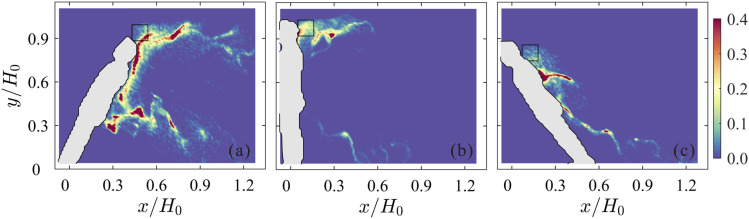
Instantaneous relative local concentration of virtual particles in the wake of the manikin with inclination angles of 60° (a), 90° (b), and 120° (c). The virtual particles were continually released into the unsteady flow fields from the initial region indicated by the black square.

From the dye visualization shown in Fig. 3, we observed that the particles in the descending case (the manikin leans backward) are advected for a long horizontal distance at the height of the head. The fluorescent particles have a density ∼1.5 times that of water. In contrast, the ratio of the density of respiratory droplets to that of air is ∼830 (at 20°C), which is unachievable in a water tunnel experiment. To circumvent this difficulty, a virtual particle simulation was carried out to capture the particle motions using the measured velocity fields. The density ratio between the virtual particles and water was set to be the same as that of the liquid droplets in air. The motion of the virtual particles is determined by the Stokes number *St*, which is a dimensionless number defined as the ratio of the characteristic time of a droplet (or a particle) to the characteristic time of the flow. *St* is defined as

$$St=\frac{\rho_{p}D_{p}^{2}}{18\mu }\cdot \frac{U_{0}}{L_{0}},$$ (1)

where *ρ*_*p*_ and *D*_*p*_ represent the density and the diameter of particles (or droplets), respectively, *μ* is the dynamic viscosity of the fluid, and *U*_0_ and *L*_0_ are the characteristic velocity and the length of the flow, respectively. When *St* ≪ 1, the particles closely follow fluid flows. In this study, the free-stream velocity and the height of the body are utilized as the characteristic velocity and length, respectively. The Stokes number of the particles in the water experiments should be equal to that of droplets in air, and consequently, the diameter of the particle *D*_*p*_ ≈ 0.13*D*_*d*_, where *D*_*d*_ represents the diameter of the droplets, which follows a Weibull distribution (Dbouk and Drikakis, 2020a). The particles were continually released into unsteady flow fields with random initial positions in a black box, as shown in Fig. 4. According to Dbouk and Drikakis (2020a), droplets with a total number of 1008 are ejected by mouth for 0.18 s, and the average droplet release rate is 8400/s, which is also used in our simulation. The motion of a virtual particle is determined by Newton’s second law, where gravity, buoyancy, and water drag force are considered in our Lagrangian solver. The drag is extracted from the velocity data of the 2D PIV measurements. The details of the simulation method can be found in Wang *et al.* (2020a). Spline interpolation of the PIV data was adopted to obtain the velocity at subgrids. When the positions of particles are determined at each time step, a box counting method is utilized to obtain the local concentration (Aliseda *et al.*, 2002). The measurement domain is divided into small boxes, and the particle number is counted in each box. The relative local concentration *C*_*r*_ is computed as *N*_*b*_/max(*N*_*b*_), where *N*_*b*_ is the particle number in each box.

Figure 4 shows the relative local concentration of virtual particles at a time instant. The left, middle, and right panels display the results for inclination angles of 60°, 90°, and 120°, respectively. In Fig. 4(a) (corresponding to the case of going downstairs), a large number of particles are distributed behind the head within an elongated region, and other particles are mainly concentrated in the region behind the waist. In Fig. 4(c) (corresponding to the case of going upstairs), most particles reattach to the back of the manikin. The distribution pattern of the virtual particles is analogous to that from the 3D RANS results of Li *et al.* (2021b). This analogy suggests that our virtual particle simulation using 2D PIV velocity data captures the key feature of the flow in this problem. More importantly, virtual particles are observed to preferentially localize in specific regions at high concentrations. They are expected to be in the regions of low vorticity and high strain rate (Aliseda *et al.*, 2002). During the advection of the particles, the particles are clustered to locally increase the concentration of virus, which leads to a higher risk of infection.

**FIG. 5.**
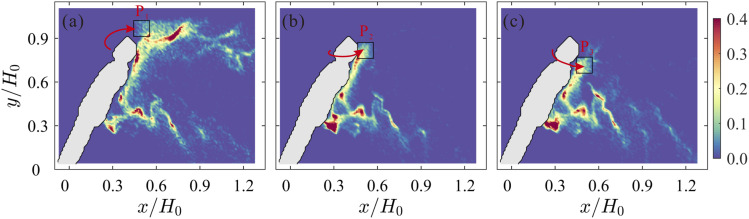
Instantaneous relative local concentration of virtual particles in different initialized locations. The positions of virtual particles are randomly initialized within the black boxes higher than the head (a), behind the head (b), and lower than the head (c). From left to right, the positions of the black boxes are indicated by *P*1, *P*2, and *P*3, respectively. The red arrows indicate the direction of motion of particles ejected from the mouth. The inclination angle of the manikin is 60°, corresponding to the case of going downstairs.

Based on the previous analysis, a person going downstairs can cause a higher infection probability for a following person due to the “upwash” velocity. It is important to study how to prevent or weaken the transport of droplets (particles). Therefore, we initialize the virtual particles at different positions to investigate the influence of position on the motion of particles. Figure 5 shows the instantaneous relative local concentration of virtual particles in different initialized locations. From left to right, the particles are randomly initialized within the regions of *P*1 (higher than the head), *P*2 (behind the head), and *P*3 (lower than the head). It is obvious that the particles higher than the head can travel a long horizontal distance at the height of the body, while the particles lower than the head move toward the ground because of the induction of the wake. These results suggest that we should cough with the head down toward the ground to ensure that most of the droplets enter the wake region. This posture may be the simplest and most efficient way to reduce droplet transmission when there is no tissue to cover the mouth and nose.

## IV. DISCUSSION AND CONCLUSIONS

In this study, the dispersion of cough-generated droplets from a person going up- or downstairs is investigated through laboratory experiments in a water tunnel, where manikins with different inclination angles are used to mimic the person. A virtual particle simulation based on the velocity data from the water tunnel experiments is also adopted to study the particle dispersion.

The flow behind a person is analyzed in more detail using PIV measurements. For a person walking up- or downstairs (corresponding to inclination angles of 120° and 60°, respectively), a “downwash” wake flow and an “upwash” wake flow are observed, causing dramatically different particle dispersion patterns. These two distinct features generally agree with the RANS results of Li *et al.* (2021b), where the steady flow field is used in their simulation of particle dispersion. Fluorescent dye injected from the mouth is also adopted to visualize the particle distribution. Induced by the flow field, the fluorescent particles indeed move toward the ground for an ascending person, while the fluorescent particles travel over the head for a descending person. The key difference for our experiments is that the particles (or droplets) disperse in the manner of particle clustering, which is caused by flow unsteadiness. Particle parcels at high concentrations may result in higher risks of infection for people following behind. Additionally, from the virtual particle simulation, we found that the motion of the particles is mainly determined by the initial position. The particles can travel a long horizontal distance at the height of the head if the initial position is higher than the head; otherwise, most of the particles move toward the ground or the back of the person. This result is consistent with the simulations by Khosronejad *et al.* (2020), where the droplets escaping from the mask are entrained by the traveling ambient flow near the top of the head and travel many meters from the person.

Based on the findings in this study, we make the following recommendations for respiratory infectious diseases. First, greater social distance is suggested for people going downstairs or riding descending escalators (Li *et al.*, 2021b), with consideration of the environment and scenario. Second, a proper and polite coughing posture is essential to prevent airborne transmission. The World Health Organization suggests covering the mouth and nose with a bent elbow or tissue when coughing or sneezing (World Health Organization, 2021). It is also critical that a person on an escalator cough or sneeze with the head down toward the ground so that the dispersed droplets (escaping from the blockage of the elbow or the tissue) are entrained into the wake region toward the ground. This may reduce the probability of droplets being inhaled by a person following behind. Third, in contrast to droplet dispersion in the steady flow field, unsteady fluctuations of the airflow can result in the clustering of droplets. A high concentration of droplets slows down the dispersion of the droplets toward a safe local concentration level and correspondingly increases the distance of droplet dispersion with safety concerns. This nontrivial effect should be considered in evaluating the dispersion of droplets in real cases.

## Data Availability

The data that support the findings of this study are available from the corresponding author upon reasonable request.
